# Elastosis Perforans Serpiginosa Is Not a Cutaneous Manifestation of Marfan Syndrome

**DOI:** 10.7759/cureus.87904

**Published:** 2025-07-14

**Authors:** Philip R Cohen

**Affiliations:** 1 Dermatology, University of California, Davis Medical Center, Sacramento, USA; 2 Dermatology, Touro University California College of Osteopathic Medicine, Vallejo, USA; 3 Maples Center for Forensic Medicine, University of Florida College of Medicine, Gainesville, USA

**Keywords:** connective tissue, cutaneous, elastosis, fbn1 gene, marfan, perforans, serpiginosa, skin, striae, syndrome

## Abstract

Elastosis perforans serpiginosa is a perforating dermatosis that morphologically presents as serpiginous, annular, or curved papules and plaques whose pathologic examination demonstrates the transepidermal elimination of elastic fibers. The perforating dermatosis can be idiopathic or induced by drug exposure: most commonly, D-penicillamine. Occasionally, elastosis perforans serpiginosa is associated with a connective tissue disease. Individuals with Marfan syndrome have a defect in the *FBN1* gene, which produces fibrillin. The connective tissue disease can occur spontaneously or is usually inherited in an autosomal dominant manner. Major criteria for the diagnosis of Marfan syndrome include aortic root dilatation and ectopia lentis. Patients often have musculoskeletal abnormalities, other cardiovascular features, and/or other ocular manifestations. A common cutaneous manifestation of Marfan syndrome is striae distensae on the deltoid, pectoral, and/or thigh regions. Numerous textbooks and publications have stated that elastosis perforans serpiginosa is associated with Marfan syndrome. However, the bona fide coexistence of elastosis perforans serpiginosa and Marfan syndrome has only been documented in a 23-year-old woman. She not only had biopsy-confirmed elastosis perforans serpiginosa but also skeletal abnormalities (arachnodactyly, genu valgum, and kyphoscoliosis) and numerous eye findings of Marfan syndrome. An investigation using the medical search engine PubMed for “elastosis perforans serpiginosa and Marfan syndrome” does not yield any relevant citations. Therefore, to the best of my knowledge, the literature only contains the coincidental observation of elastosis perforans serpiginosa and Marfan syndrome in a single patient. In conclusion, elastosis perforans serpiginosa is not a cutaneous manifestation of Marfan syndrome.

## Editorial

Elastosis perforans serpiginosa is an infrequently occurring perforating dermatosis that may be idiopathic, drug-induced (most often related to D-penicillamine), or associated with a connective tissue disease; it was initially referred to as Miescher’s elastoma (elastoma intrapapillare perforans verruciforme) [1]. Clinically, elastosis perforans serpiginosa is characterized by papules or plaques that are arranged in a serpiginous, annular, or curved distribution that are most commonly found on the face, neck, arms, and flexural areas [2]. Microscopic examination demonstrates the transepidermal elimination of elastic fibers [2].

Marfan syndrome is an autosomal dominant connective tissue disease that results from a defect in the *FBN1* gene, which is located on chromosome 15 and produces the connective tissue protein fibrillin. Major criteria for the diagnosis of Marfan syndrome include aortic root dilatation and ectopia lentis. Additional clinical features include musculoskeletal abnormalities, other cardiovascular disorders, and other ocular manifestations. Striae distensae are a characteristic cutaneous feature and are typically located on the deltoid, pectoral, and/or thigh regions [3].

Numerous textbooks and publications have stated that elastosis perforans serpiginosa is associated with Marfan syndrome [3]. It is likely that Mehregan’s 1968 landmark review on elastosis perforans serpiginosa prompted clinicians and future investigators to accept the correlation of the two conditions as dogma [2]. In his paper, Mehregan reported 11 new cases of elastosis perforans serpiginosa and summarized the observations of the previously described 90 cases [2]. The paper identified that 26% of the cases had a systemic disorder; it also claimed that two of the elastosis perforans serpiginosa patients possibly had Marfan syndrome [2,4,5].

One of the patients Mehregan considered to have Marfan syndrome was an 18-year-old man with elastosis perforans serpiginosa [2]. The man, reported by Anning, died following the acute onset of a dissecting aortic aneurysm [5]. Several subsequent investigators did not confirm the diagnosis of Marfan syndrome in this patient [1,3].

However, there is only a single previously reported patient in whom elastosis perforans serpiginosa and Marfan syndrome co-existed [4]. In 1952, a 23-year-old woman with Marfan-type arachnodactyly was reported by Storck [4]. Numerous researchers concurred that the woman had Marfan syndrome [1-3].

The woman’s skin condition was originally described as hyperkeratosis follicularis et perifollicularis in cutem penetrans (Kyle’s disease) [4]. However, a subsequent pathology review of her skin lesion biopsy by Dr. Miescher concluded that they were Miescher’s elastoma, and therefore, elastosis perforans serpiginosa [1].

Although the diagnostic criteria for Marfan syndrome had not been established in 1952, the 23-year-old woman had numerous clinical stigmata consistent with the diagnosis of Marfan syndrome: arachnodactyly, genu valgum (knock knees), kyphoscoliosis, and numerous eye findings (divergent strabismus, astigmatism, and keratoconus) [1-4]. In addition, the subsequent review of the pathology from her skin lesions established the concurrent diagnosis of elastosis perforans serpiginosa [1].

To the best of my knowledge, the literature only contains a single Marfan patient with elastosis perforans serpiginosa [4]. An investigation using the medical search engine PubMed for “elastosis perforans serpiginosa and Marfan syndrome” did not yield any relevant citations. In conclusion, although the coincidental observation of elastosis perforans serpiginosa and Marfan syndrome has been described in a single individual, elastosis perforans serpiginosa is not a cutaneous manifestation of Marfan syndrome.
